# ‘Shadow’ glass transition in glass

**DOI:** 10.1093/nsr/nwab160

**Published:** 2021-08-26

**Authors:** Yuanzheng Yue

**Affiliations:** Department of Chemistry and Bioscience, Aalborg University, Denmark

The glass transition is the most striking dynamic feature of glass-forming liquids and rigid glass, manifest in the following two scenarios [1]. First, a viscous glass-forming liquid is transformed into a rigid glass state during cooling if the cooling rate is high enough to prevent the liquid from crystallizing—this process is called the ‘liquid–glass transition’. Second, a rigid glass state is transformed into a viscous liquid on heating—this process is called the ‘glass–liquid transition’ or simply the ‘glass transition’. Substantial progress has been made in understanding of the glass transition through atomistic simulations and advanced structure probing tools [1]. However, despite such progress, there is much we do not know about the glass transition, largely because of difficulties in probing the structural response of liquid or glass to the glass transition at different length and time scales. Thus, the nature of glassy state was selected as one of the 125 big scientific questions of our time by *Science* in 2005, where it was asked ‘Where and why does liquid end and glass begin?’. This question continues to attract considerable interest of scientists.

As a crucial contribution to understanding of the calorimetric glass transition, several groups of scientists independently observed a striking pre-endothermic event, that is an endothermic pre-peak before the real glass transition starts. This phenomenon occurs in annealed hyperquenched (HQ) (cooled at >10^5^ K/s) glasses including metallic [2], organic [3] and inorganic systems [4], respectively, during dynamic reheating (or upscan) in a differential scanning calorimeter (DSC). The pre-peak is followed by an exothermic sub-*T_g_* peak in HQ glass that has undergone a certain duration of annealing below *T_g_*. The reheating and the subsequent cooling are conducted at the standard rate of 20 K/min. The glass cooled at this rate is usually defined as the ‘standard’ glass. The cooling rate for a HQ glass is more than six orders of magnitude higher than the standard rate. The magnitude of the SGT peak is quantified by the difference in isobaric heat capacity (*C_p_*) between the annealed HQ glass and the standard glass in the temperature range of the SGT peak, and it is expressed as Δ*C_p_*_@_*_Tg__,__shadow_*. To distinguish from the real reversible glass transition peak, the endothermic pre-peak was expressed as the shadow glass transition (SGT) by Yue and Angell [4]. Both the magnitude and temperature of the SGT peak continuously increase with increasing annealing temperature and time.

As a significant step towards understanding of SGT, Yang *et al.* recently detected SGT peaks in HQ metallic glasses (MGs) using the chip-based fast scanning calorimetry (FSC) without performing annealing [5]. In doing so, they verified that SGT is a universal feature of HQ metallic glasses. They elegantly revealed the quantitative correlation of SGT with β relaxation by comparing the calorimetric traces with dynamic mechanical spectra (DMS). Figure 1a shows the pronounced endothermic SGT peaks for three HQ metallic glass systems, which are followed by the exothermic enthalpy relaxation peaks, and then by the real glass transition peaks. For the Pd_40_Ni_40-_*_x_*Cu*_x_*P_20_ glass, a crystallization peak appears subsequent to the glass transition. As illustrated in Fig. 1a, the magnitudes of both SGT and real glass transition are determined by the heights of their corresponding peaks, that is Δ*C_p_*_@_*_Tg__,__shadow_* and Δ*C_p_*_@_*_Tg_*, respectively. Figure 1b displays the direct correspondence between SGT and β relaxation in the same temperature range through the FSC and DMS measurements, while Fig. 1c shows the proportionality between SGT and β relaxation. These results thus become the first direct evidence for the dynamic origin of SGT. The evidence is supported by the heating rate dependences of both SGT and β relaxation overlapping with each other, and merging with those of the real glass transition and α relaxation at sufficiently high temperature, that is well above the standard *T_g_*. This implies a bridge between the dynamics and the thermodynamics of β relaxation. Interestingly, Yang *et al.* also discovered that the SGT temperature (*T_g__,__shadow_*) first decreases with quenching rate, and then remains unchanged when the quenching rate exceeds 10^6^ K/s. This indicates that a critical *T_g__,__shadow_* value exists, below which the glass structural domains with the lowest potential energy cannot be rejuvenated to the average energy level of the standard glass.

**Figure 1.**
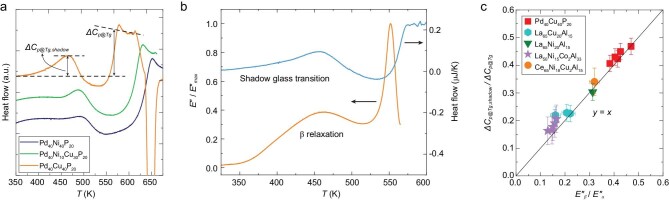
Coupling between shadow glass transition and β relaxation [5]. (a) FSC heat flow curves for Pd_40_Ni_40-_*_x_*Cu*_x_*P_20_ (*x* = 0, 30 and 40) MGs at an upscan rate of 500 K/s, showing quantification of the extents of both SGT (Δ*C_p@Tg__,__shadow_*) and real glass transition (Δ*C_p@Tg_*). (b) FSC heat flow (200 K/s) versus DMS loss modulus (2 Hz). (c) Relationship between the normalized extent of SGT (Δ*C_p@Tg__,__shadow_/*Δ*C_p@Tg_*) and that of β relaxation (*E’’*_β_/*E’’*_α_).

In summary, the work by Yang *et al.* provides a great advancement in revealing the nature of the shadow glass transition. Their findings have implications for the connection between the shadow and real glass transitions, as well as for correlation of the shadow glass transition with both β relaxation and physical properties of hyperquenched glasses [6]. Their work is anticipated to stimulate research into the microscopic origin of the shadow glass transition.

***Conflict of interest statement*.** None declared.

## References

[1] Debenedetti PG , Stillinger FH. Nature 2001; 410: 259–67. 10.1038/35065704 11258381

[2] Chen HS , Inoue A. J Non-Cryst Solids 1984; 61&62: 805–10. 10.1016/0022-3093(84)90641-0

[3] Berens AR , Hodge IM. Macromolecules 1982; 15: 756–61. 10.1021/ma00231a015

[4] Yue YZ , Angell CA. Nature 2004; 427: 717–20. 10.1038/nature02295 14973480

[5] Yang Q , Peng SX, Wang Z et al. Natl Sci Rev 2020; 7: 1896–905. 10.1093/nsr/nwaa100 34691531 PMC8288642

[6] Yang Q , Pei CQ, Yu HB et al. Nano Lett 2021; 21: 6051–6. 10.1021/acs.nanolett.1c01283 34240612

